# Grain flash temperatures in diamond wire sawing of silicon

**DOI:** 10.1007/s00170-021-07298-7

**Published:** 2021-06-11

**Authors:** Uygar Pala, Stefan Süssmaier, Konrad Wegener

**Affiliations:** 1 Inspire AG, Technoparkstrasse 1, 8005 Zurich, Switzerland; 2 Institute of Machine Tools and Manufacturing (IWF), ETH Zurich, Leonharstrasse 21, 8092 Zurich, Switzerland

**Keywords:** Flash temperature, Silicon, Wire sawing, Single-grain scratching, Wear

## Abstract

Diamond wire sawing has obtained 90% of the single-crystal silicon–based photovoltaic market, mainly for its high production efficiency, high wafer quality, and low tool wear. The diamond wire wear is strongly influenced by the temperatures in the grain-workpiece contact zone; and yet, research studies on experimental investigations and modeling are currently lacking. In this direction, a temperature model is developed for the evaluation of the flash temperatures at the grain tip with respect to the grain penetration depth. An experimental single-grain scratch test setup is designed to validate the model that can emulate the long contact lengths as in the wire sawing process, at high speeds. Furthermore, the influence of brittle and ductile material removal modes on cutting zone temperatures is evaluated.

## Introduction

In mechanical material removal, the highest share of the input energy is transformed to thermal energy [1]. Kennedy [2] states that 95% of the energy dissipation happens in the top of the bodies in contact, and according to Kato [3], virtually all of this energy is converted to heat at the grain-workpiece interface. The energy created during the short contact time leads to high temperatures at the grain, called the flash temperatures [4]. In grinding, the time of contact of a grain with the workpiece is in the range of 1 μs; hence, the interaction can be defined as near-adiabatic. However, in diamond wire sawing, the presence of long contact lengths emphasizes the influence of temperature effects.

Diamond wire sawing is a process predominantly used to slice hard and brittle material into wafers. In recent years, wire sawing with fixed grains has evolved to replace wire sawing with slurry due to advantages like lower kerf loss, higher material removal rate, and superior wafer strength [5]. Single-crystal silicon (Si) wafers for semiconductor and photovoltaic applications are typical applications that feature very long contact length of traditionally 156 mm, while the trend for solar cells is to increase the wafer size to 210 mm and possibly up to 300 mm in the future.

Temperatures in the cutting zone have great influence on the grain wear. Wear models such as those proposed by Takeyama and Murata [6], Usui et al. [7], and Jiang and Arnell [8] predict a dependence of wear on the contact temperature. During turning of Si with diamond tools, carbon diffuses into the Si surface to form hard silicon carbide (SiC), which consequently leads to heavy tool wear on the flank face [9]. The formation of SiC and diamond-like carbon structures on the Si surface is accelerated by high temperature and pressure. Formation of SiC was not observed in diamond scratching with an engagement length of 20 mm, a depth of cut of 10 μm, and a low cutting speed of 100 mm/min [10]; a possible explanation lies in the interruption of the diffusion process with brittle fracture and the short contact length. Analysis of worn wires used to cut 156-mm Si ingots with 15 m/s showed graphitic phases on 3 out of 21 diamonds analyzed [11].

**Table 1:** Nomenclature used in the study (formula symbols only)

Symbol	Meaning	Unit
*κ*_*w*_	Thermal diffusivity of silicon	[*m*^2^/*s*]
*ρ*_*g*_	Material density of diamond	[*kg*/*m*^3^]
*ρ*_*w*_	Material density of silicon	[*kg*/*m*^3^]
*A*_⊥_	Orthogonal grain area normal to the workpiece surface	[*μm*^2^]
*A*_*cu*_	Penetrated grain area projected into direction of cut	[*μm*^2^]
${C_{p}^{g}}$	Specific isobaric heat of diamond	[*J*/*kgK*]
${C_{p}^{w}}$	Specific isobaric heat of silicon	[*J*/*kgK*]
*F*_*c*_	Cutting force	[*N*]
*h*_*cu*_	Grain penetration depth, undeformed chip thickness	[*μm*]
*h*_*g*_	Grain height	[*μm*]
*k*_*c*_	Specific cutting force (empirically determined)	[*N*/*mm*^2^]
*k*_*b*_	Thermal conductivity of nickel (bonding material)	[*W*/*mK*]
*k*_*g*_	Thermal conductivity of diamond	[*W*/*mK*]
*k*_*w*_	Thermal conductivity of silicon	[*W*/*mK*]
*l*_*g*_	Grain length	[*μm*]
*m*_*c*_	Cutting force model exponent (empirically determined)	[−]
*Pe*	Peclet number	[−]
$\dot {q}_{g}$	Heat flux to the grain	[*W*/*m*^2^]
$\dot {q}_{t}$	Total heat flux	[*W*/*m*^2^]
$\dot {q}_{w}$	Heat flux to the workpiece	[*W*/*m*^2^]
*t*	Time	[*s*]
*T*	Temperature	[*K*]
*T*_*b*_	Temperature of the bonding material	[*K*]
*T*_*f*_	Flash temperature at the grain tip	[*K*]
*v*_*c*_	Cutting speed	[*m*/*s*]
*v*_*rel*_	Relative sliding velocity	[*m*/*s*]
*w*_*g*_	Grain width	[*μm*]

The workpiece surface quality is also affected by the cutting temperature. While it was shown that the coolant has a significant effect on the wafer temperature field [12], temperatures in the contact zone are typically much higher than the boiling temperature and therefore have little effect on the temperature in the cutting zone [13]. Lindholm et al. [14] have studied the coolant temperature rise during wire sawing of multi-crystalline silicon and have simulated the temperature change during the cut along with the resulting effects of thermal expansion. Depending on the cooling conditions, the heat generation between the wires and the silicon block has been determined to lie between 0.28 and 0.43 W/mm, leading to temperatures in the material of up to 270 ^∘^C in the beginning of the cut. Thermal deflection of the wafer along the wire direction of up to 10 μm may have a deteriorating effect on the total thickness variation of the wafer.

Temperature measurement in grinding processes is generally possible by measuring conducted or radiated heat [15], where methods using heat conduction typically apply thermocouples and methods measuring radiation use infrared sensors, often in combination with fibers. Thermocouples are mainly integrated into the (stationary) workpiece and an average temperature is measured; however, single-grain contact detection is not possible. Temperatures of single-grain contacts were first captured applying an infrared radiation pyrometer by Ueda et al. [16] who thoroughly analyzed the method and stated it was suitable for measuring highly dynamic temperatures of small objects. A single-grain scratching setup for measurement of the grain flash temperature during cutting of Ti6Al4V with a diamond, in which the fiber was placed below the diamond and therefore measured temperature conducted or radiated through the gain, was recently introduced [17]. The grains are brazed onto a pin and are significantly larger than those applied in diamond wire sawing.

Only a few publications with relevance to modelling the flash temperature in grinding are available. An approach for modelling temperatures during grinding is based on kinematic conditions that showed satisfactory agreement with experiments [18] but considered the temperature of the workpiece and is therefore much lower than the flash temperature observed in this study. Modelling the flash temperature of single-grain scratching by means of finite element models in 2D [19] and the chip flash temperature in 3D [20] showed good agreement with respectively analytical and experimental results. The modelling approach taken in this study builds upon the work of Archard [4], Carslaw and Jaeger [21], and Jaeger [22] which are presented in Section 3.

To date, there are no experimental setups, results, or analytic models available for the thermal characterization of the contact zone in the wire sawing process. This work presents a novel experimental setup, designed to conduct high-speed grain scratching experiments with long contact lengths and measurement of process forces and flash temperatures in the cutting region along with the respective measurement methods. Furthermore, a flash temperature model is presented with its experimental validation and the results are discussed. The model relates flash temperatures with geometric grain-workpiece contact and sliding velocity. It can therefore be used to estimate temperatures for wear modelling when using a kinematic modelling approach to determine the interaction between grain and workpiece. Formula symbols used to derive the model are presented in Table 1.

## Experimental setup and method

**Fig. 1:**
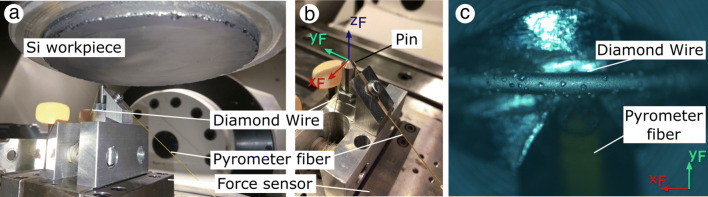
**a** Overview of the scratch test setup. **b** Details of the pin and the position of the wire. **c** Alicona IFM image of the isolated grain on pin tip and fiber of the pyrometer

The overview of the scratch test setup is presented in Fig. 1a and details of the tool are shown in Fig. 1b where the machine axes and position of the two-color fiber pyrometer are identified. The workpiece is rotating with an angular velocity *ω* and the tool is mounted on a force measurement platform and is stationary. The diamond wire is positioned and guided in the groove at the tip of the aluminum pin and is fixed at both ends. A single grain on the diamond wire is isolated at the top of the pin which has a tip radius of 0.5 mm. In Fig. 1c, a microscope image of the wire and pyrometer fiber is shown.

The setup is mounted on a Fehlmann Versa 825 5-axis milling machine. This machine fulfills requirements regarding stiffness and spindle speed required for the conduction of the experiment; the HSK63 spindle interface is suitable to hold an adapter clamping the heavy Si workpiece and safely fixing it at high spindle speeds. The force measurement system is composed of a Kistler MiniDyn Type 9256C1 3-component force dynamometer, Kistler Type 5080A charge amplifier, National Instruments NI 9222 analog-to-digital converter, and a measurement laptop with Labview software. A Fire-3 two-color fiber optic pyrometer is used to measure the temperatures. No coolant is applied. Optical measurement of the grain and the workpiece surface are performed using an Alicona Infinite Focus G4 microscope (IFM).

**Fig. 2:**
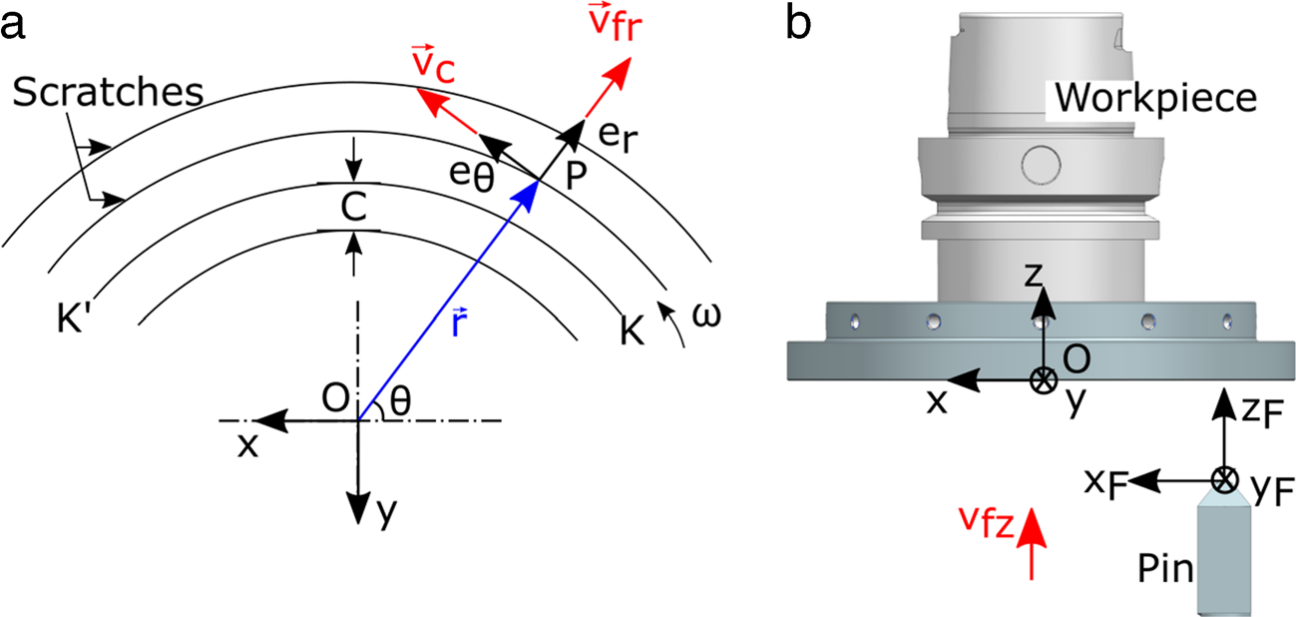
**a** Scratch paths on the workpiece surface viewed from the bottom of the workpiece. **b** Motion of the grain cutting edge

The cutting kinematics and the coordinate system are defined in Fig. 2. The contact is described from the viewpoint of point P on the grain tip which is assumed to be moving over the cutting path instead of workpiece rotation. Both approaches represent the identical kinematic conditions, while the former serves simplicity of the explanation. The grain starts its motion with zero penetration at point K and moves with an angular velocity *ω* in clockwise direction. The grain is advanced onto the surface with the vertical feed rate *v*_*fz*_; and the radial feed rate *v*_*fr*_ dictates the distance C between two consequent scratches.

**Fig. 3:**
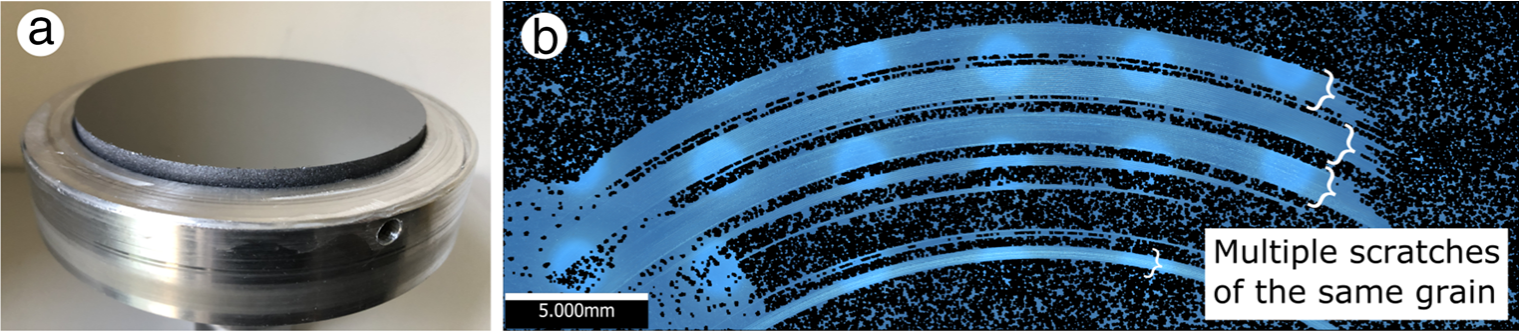
**a** Mirror-like surface of the SGT workpiece obtained through several lapping and polishing steps. **b** Scratched surface sections from several cuts

Figure 3a shows the lapped and polished, mirror-like Si workpiece surface with a maximum profile height of *R*_*z*_ = 0.49 − 0.52 μm determined in accordance with DIN EN ISO 4287 [23]. The material used is commercial solar-grade monocrystalline Si. In Fig. 3b, an Alicona IFM image of multiple scratches on the workpiece surface is presented. Each identified section shows passes by the same grain and consists of several consequent scratches with increasing penetration depth. The exact penetration depth cannot be controlled because of a slightly irregular concave shape of the workpiece surface. The penetrated depth or undeformed chip thickness *h*_*cu*_ is estimated from the residual scratch depth according to the methods described in [24]. A continuous contact between the grain and Si workpiece is not possible due to a small perpendicularity error of the workpiece surface with the spindle axis; instead, a section of the surface is scratched where the contact length of one grain pass is 40 mm and the average total contact distance of a grain is 0.8 m. The cutting time is determined from the measured force signal. Both ductile and brittle material removal can be observed and associated with the respective forces and temperatures.

**Fig. 4:**
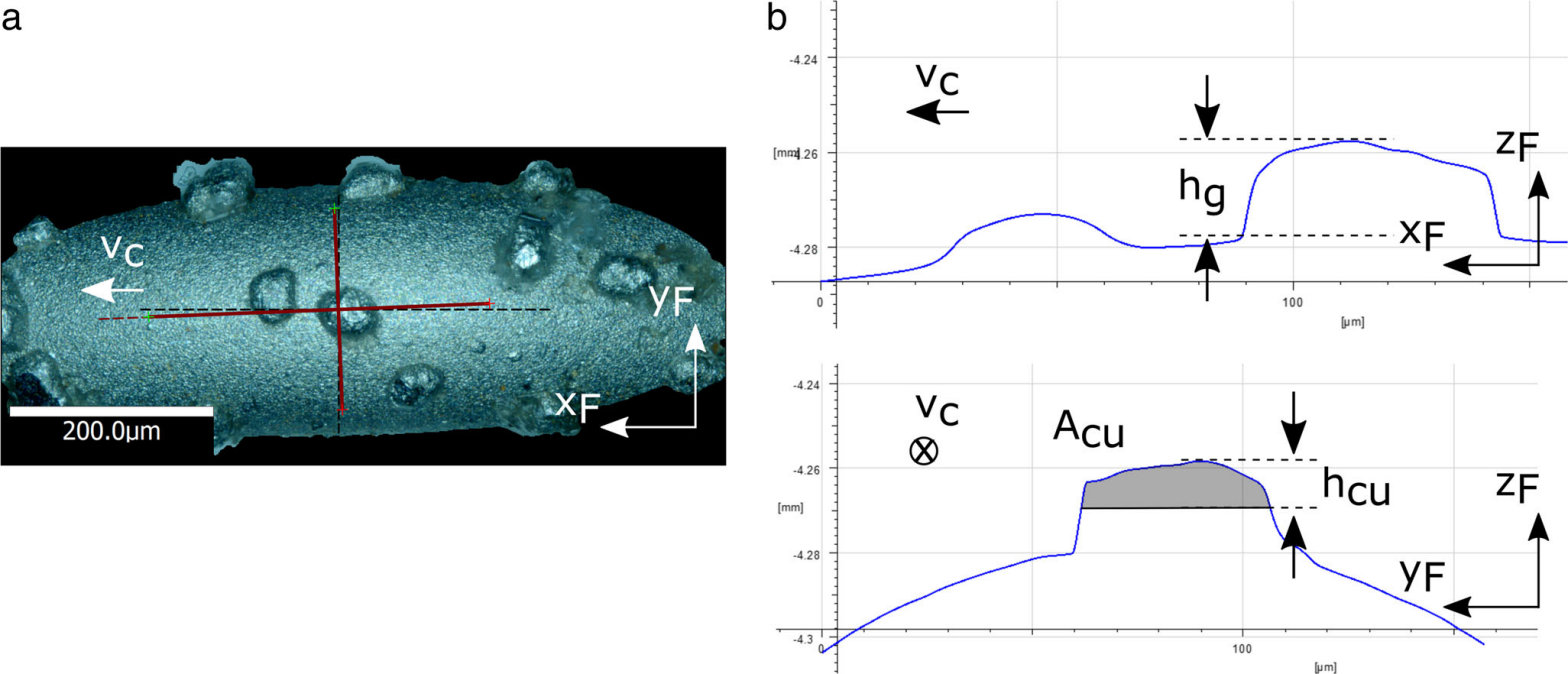
**a** Alicona IFM image of an abrasive grain on pin tip. **b** Grain cross sections orthogonal and normal to the cutting direction

Figure 4a shows the top view of the Alicona IFM image of an isolated abrasive grain on the pin tip. Figure 4b shows the grain cross sections parallel and orthogonal to the cutting direction. The grain protrusion *h*_*g*_ is measured from the surface of the wire. The grain penetration depth of *h*_*cu*_ and cutting area *A*_*cu*_ are shown. The grain wears over the course of the experiment, and its protrusion decreases by 0.5–1.5 μm. The ratio of cutting area to penetration depth *A*_*cu*_/*h*_*cu*_ is more or less constant when comparing the grain before and after the cut, implying that the grain wears out regularly in terms of the shape projected into the cutting plane, even though the cutting edge rounds off during the cut. The typical wear behavior of a single diamond grain in contact with Si has been analyzed in the past [25].

**Fig. 5:**
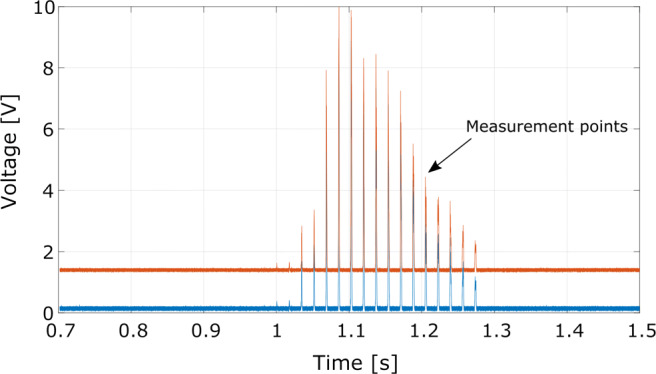
Voltage readings from two pyrometer wavelengths

For this work, flash temperatures at the grain tips that are measured during the scratch tests are evaluated. Flash temperatures from a series of scratches performed with individual grains are measured. Figure 5 shows the voltage readings of two different wavelengths where each peak corresponds to a single scratch. The beginning and end of the contact are identified from the voltage signal; a threshold value of 0.1 V is applied.

**Fig. 6:**
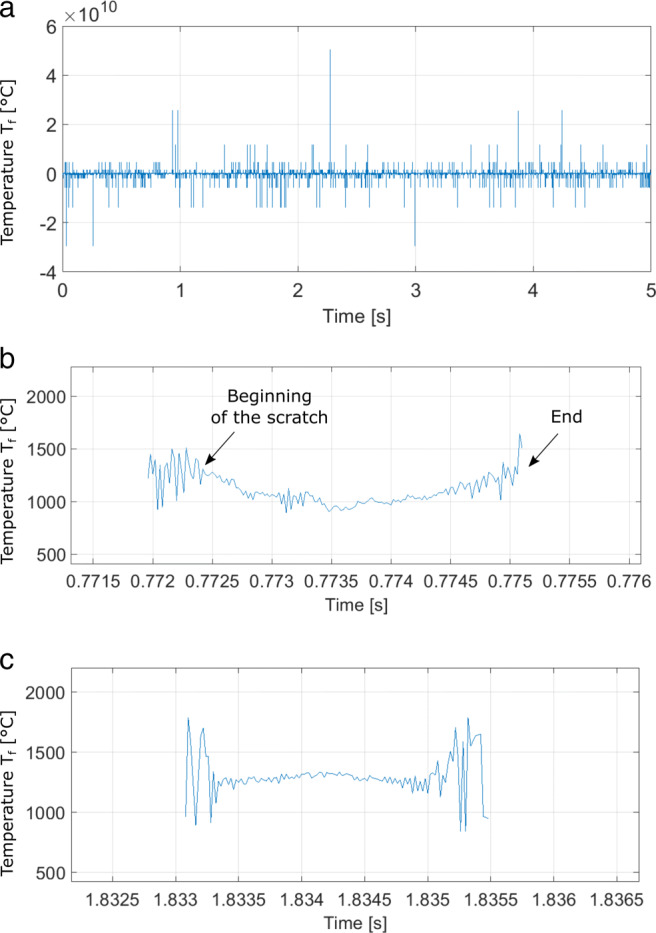
**a** Plot of the raw temperature data. **b** Measured temperatures of an individual brittle scratch. **c** Measured temperatures of an individual ductile scratch

The temperature values corresponding to the presented wavelengths are shown in Fig. 6. The raw measurement data is presented on top, where a very high noise-to-signal ratio necessitates the definition of the beginning and end of the scratch from the voltage signal. Figure 6b and c show isolated and typical temperature readings associated with a brittle (b) and ductile (c) scratch.

## The flash temperature model

The problem of flash temperatures at the grain tip can be described as the distribution of the energy produced in the contact area through relative sliding of the grain and workpiece. The total heat generated *Q*_*t*_ is distributed as *Q*_*g*_ conducted to the grain, *Q*_*w*_ to the workpiece, *Q*_*ch*_ to the chip, and *Q*_*f*_ to the coolant. However, the share of heat extracted by the chips is limited [26] and much smaller in comparison to the heat conduction into the grain and workpiece; hence, *Q*_*ch*_ can be neglected. Moreover, in the contact region, the temperatures are far higher than the coolant boiling temperature, limiting the convection effects in the cutting zone [13]. The effect of the cooling fluid is hence restricted to cooling of the tool outside the contact region [27]. The total heat rate generated per unit area $\dot {q}_{t}$ is then shared between the grain and workpiece:

1 $$ \dot{q}_{t} = \dot{q}_{g} + \dot{q}_{w} $$

where $\dot {q} = {Q_{t}}/{A_{\perp }}$, and *A*_⊥_ is the orthogonal grain area in the direction normal to the workpiece surface. The total heat rate generated is:

2 $$ \dot{q}_{t} = \frac{F_{c} \cdot v_{rel}}{A_{\perp}} $$

where the cutting force *F*_*c*_ is acting on the grain parallel to the relative sliding velocity *v*_*rel*_.

**Fig. 7:**
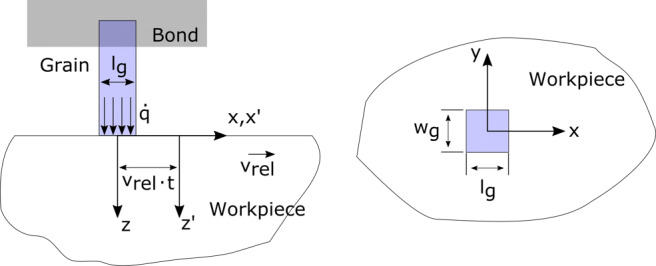
Dimensions and definitions of the grain-workpiece contact conditions

The problem is then reduced to the determination of the share of the produced heat to the workpiece and to the diamond grain. The condition on the workpiece side can be defined as a stationary heat source on a moving body, as schematically described in Fig. 7. The heat source (grain) is stationary with the origin of the fixed coordinate system (x, y, z) and the semi-infinite body is sliding at *z* = 0 with a constant velocity *v*_*rel*_ with the coordinate system (x’, y’, z’) attached to its body.

**Fig. 8:**
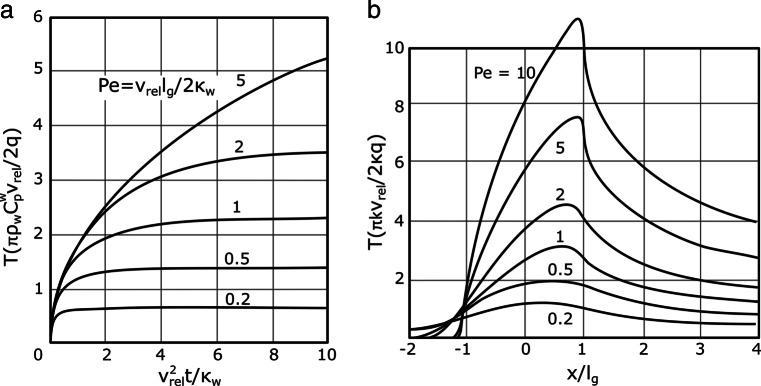
**a** Temperatures at the surface of a square heat source sliding with a constant velocity as a function of time and Peclet number [22]. **b** Temperature behavior on a semi-infinite solid, resultant of a sliding of a surface with width 2b [21]

A significant simplification of the heat conduction problem is to assume steady-state heat flow instead of a transient condition. The time frames until the steady-state conditions are reached were studied by Bhushan [28], to find that the flash temperature reaches its steady state after a sliding distance of 1.25 times the length of the source. Furthermore, for a moving circular heat source, Yevtushenko [29] stated that the temperature at the contact zone reaches 87*%* of its steady almost instantly. The transient conditions were studied by Jaeger [22] in the case of a moving square heat source over a surface. The time until steady state is reached can be described by the nondimensional Peclet number. The times at which the temperature reaches steady-state condition are plotted in Fig. 8a. The Peclet number for square and band sources is defined as:

3 $$ Pe = \frac{v_{rel} \cdot (l_{g}/2)}{2 \cdot \kappa_{w}} $$

where *l*_*g*_ is the length of the contact surface in the sliding direction, *l*_*g*_ = *w*_*g*_ is assumed in Fig. 7, and *κ*_*w*_ is the thermal diffusivity of Si, defined as:

4 $$ \kappa_{w} = \frac{k_{w}}{\rho_{w} \cdot {C_{p}^{w}}} $$

where *k*_*w*_ is the thermal conductivity, *ρ*_*w*_ is the density, and ${C_{p}^{w}}$ is the specific isobaric heat of Si.

The primary assumptions of the temperature model are:

- Contact and penetration area between the grain and the workpiece remain constant over the contact time
- Diamond and silicon have constant and temperature-independent material properties
- Heat extraction due to coolant and chip is negligible
- No heat exchange between the grain and the surroundings
- No internal heat generation, especially negligible heat generation due to plastic dissipation during chip formation
- Steady-state heat flow in the diamond
- Constant bonding material temperature

The solution for a square uniform band heat supply acting on a semi-infinite solid surface is defined by Carslaw and Jaeger [21] and the results are plotted in Fig. 8b, which are highly dependent on the Peclet number.

For *Pe* ≤ 10 and a uniform band source heat distribution, the maximum flash temperature rise *ΔT*_*f*_ is approximated by Tian [30] as:

5 $$ {\varDelta} T_{f} = \frac{2 \cdot \dot{q}_{w} \cdot (l_{g}/2)}{k_{w} \sqrt{\pi(1+Pe)}} $$

where *k*_*w*_ is the thermal conductivity of silicon, *Pe* is the Peclet number defined for the relative sliding speed, and *l*_*g*_ is the grain side length orthogonal in the direction of sliding.

One-dimensional heat conduction towards the bonding surface with constant material parameters is assumed for the grain:

6 $$ \frac{\partial T}{\partial t} = \frac{k_{g}}{\rho_{g} \cdot {C_{p}^{g}}} \frac{\partial^{2} T}{\partial x^{2}} $$

**Fig. 9:**
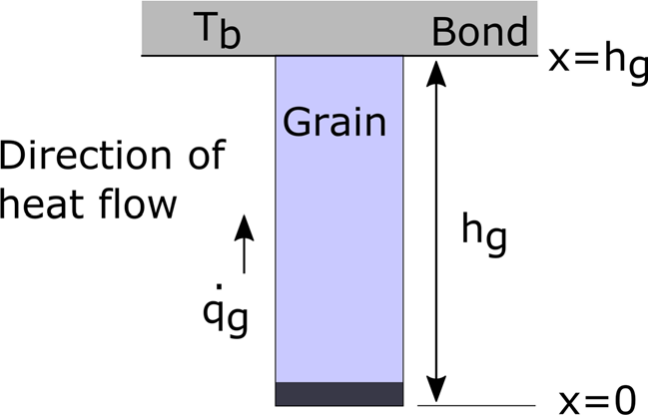
Parameters of the grain model and the direction of heat flow

It is required to define the initial and boundary conditions to solve the equation. Based on Fig. 9, in the case of a steady-state, linear, one-dimensional heat transfer in a column, the initial and boundary conditions (IC and BC respectively) can be stated as:

7 $$ \begin{array}{@{}rcl@{}} & B.C.^{1}: & \quad\text{at } x = 0  \qquad\qquad\qquad\qquad\qquad\quad{\dot{q}=-k_{g}\frac{\partial T}{\partial x}} \end{array} $$

8 $$ \begin{array}{@{}rcl@{}} & B.C.^{2}: & \quad\text{at } x = h_{g} \qquad{\dot{q}=-k_{g}\frac{\partial T}{\partial x} = -k_{b} (T_{x=h_{g}}-T_{b})} \end{array} $$

**Table 2:** Thermal properties and densities of sc-Si and Type IIa single-crystal diamond at 300 K

	sc-Si [31]	Diamond [32]
Density *ρ* [*kgm*^− 3^]	2329	3520
Specific isobaric heat *C*_*p*_ [*Jkg*^− 1^*K*^− 1^]	713	502–519
Thermal conductivity *k* [*Wm*^− 1^*K*^− 1^]	156	2000–2100

The temperature distribution as a function of distance from the grain tip is then defined as:

9 $$ T(x) = \frac{T_{b} - T_{f}}{h_{g}}x + T_{f} $$

The rate of heat flow per unit area is found:

10 $$ \dot{q}_{g} = k_{g} \frac{(T_{f}-T_{b})}{h_{g}} $$

Combining Eqs. 1, 2, 5, and 10, the flash temperature at the grain tip can be calculated:

11 $$ T_{f} = \frac{k_{c1} \cdot A_{cu}^{1-m_{c}} \cdot 2 \cdot (l_{g}/2) \cdot h_{g}}{k_{g} \cdot A_{\perp} \cdot 2 \cdot (l_{g}/2) + k_{w} \cdot A_{\perp} \cdot h_{g} \sqrt{\pi(1+Pe)}} $$

where *k*_*c*_ is the specific cutting force and *m*_*c*_ is the material-pair dependent constant, which are experimentally determined for diamond and single crystal Si (sc-Si) [24]. Properties of sc-Si and synthetic single crystal diamond are listed in Table 2.

## Results and discussion

A useful observation is presented in Fig. 6b and c, where measured temperatures of representative brittle and ductile scratches are depicted. In both graphs, the grain starts its penetration from the left side and the contact ends at the right side of the plot. The middle part of the plot shows the state at the maximum grain penetration. In the case of brittle material removal, as the penetration depth increases, the rate of fracture increases, the energy is dissipated with fracture, and lower temperatures are measured at the grain tip. In the ductile material removal case, a higher share of energy is dissipated in the form of thermal energy, leading to a temperature increase as the grain penetration increases.

**Fig. 10:**
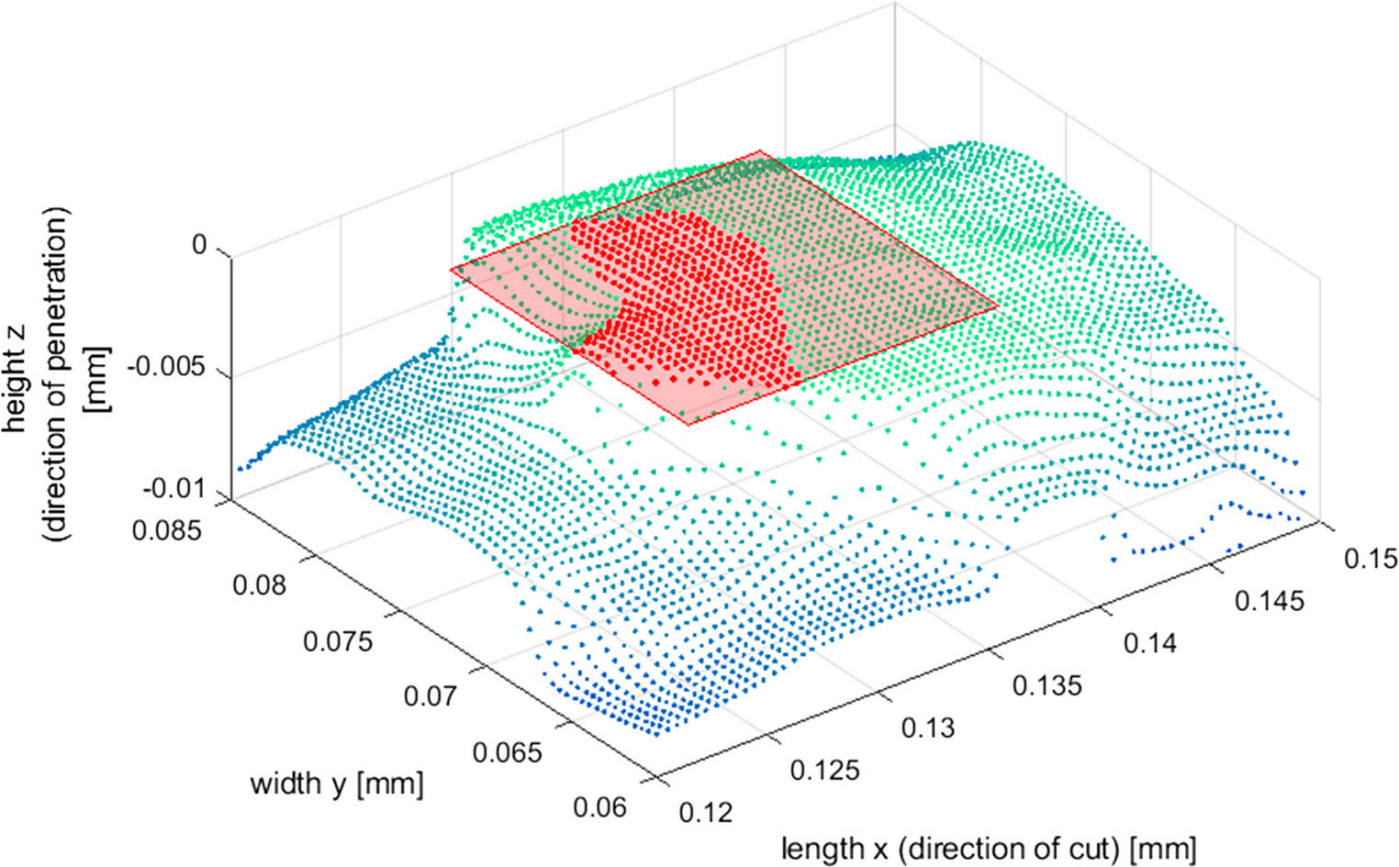
Contact area between a grain and the workpiece at a penetration depth of *h*_*cu*_ = 570nm; point cloud representation of the microscopic image of a grain used in the experiments with vertices in contact shown in red and the respective model assumption of a square contact shown as red rectangle

Model validation experiments are conducted with 6 grains and 90 scratches in total, at the cutting speed of 10 m/s. The comparisons of the simulated and measured results show that the model underestimates the flash temperature *T*_*f*_ by a factor of ≈ 4.5. While this factor may seem large, it can be explained through consideration of the most significant simplifications adopted in the model:

- The contact between the grain and the workpiece material *A*_⊥_ is not rectangular and largely overestimated in the model. Figure 10 shows a point cloud representation of a grain used in the experiments. The vertices on the grain surface that are in contact with material during a scratch are marked as red dots while the model assumption of a square contact based on the width of the grain in contact is shown as a red square. There is a significant deviation between the assumption and the real observation, where the area assumed in the model is between 1.5 and 2.5 times larger than the area based on the real contact geometry. The larger contact area *A*_⊥_ reduces the modelled flash temperature according to Eq. 11.
- $\dot {q}_{g}$ is overestimated in the model. This is due to the assumption that the temperature of the bonding *T*_*b*_ is equal to the ambient temperature, operating as a heat sink. This is clearly not the real case, where it is expected that the heat flow from the grain tip to the bonding material shall result in a background temperature rise on the wire, leading to a decrease in the temperature difference and reduction in the rate of heat flow. A reduced heat flow through the grain will increase the flash temperature.
- The assumption that all heat introduced into the diamond stems from the sliding contact is another significant deviation from reality. For this model, the total heat generated is assumed to equal *Q*_*t*_ = *F*_*c*_ ⋅ *v*_*rel*_. This relationship obviously neglects all effects of the significantly larger normal force *F*_*n*_ exerted onto the surface and therewith associated with plastic deformation. It is generally assumed that the ratio of dissipated heat to plastic work (known as the Taylor-Quinney coefficient *β*) is a constant and has a value of 0.9, while it has been shown that it depends on the material and on strain and strain rate for steels [33]. The Taylor-Quinney coefficient for Si is unknown, but since plasticity is observed during the cutting of silicon especially in ductile cutting, it is likely that it is a substantial heat source that is neglected in the model.

The flash temperature is simulated using geometric data of the grains used in the experiments. The simulated flash temperature is multiplied with a constant coefficient of 4.5 to compensate the abovementioned underestimation. Through substituting the penetration area with the integral of the grain width over the penetration depth, $A_{cu} = {\int \limits }_{0}^{h_{cu}} w_{g}(z) dz$ in Eq. 11 gives the flash temperature value as a function of the grain penetration depth *h*_*cu*_. The comparison of the measured and simulated flash temperatures is plotted in Fig. 10 as a function of the grain penetration depth. The results suggest that the flash temperature values decrease with increasing penetration, showing that grains cutting in the brittle removal regime result in slightly lower temperature values.

**Fig. 11:**
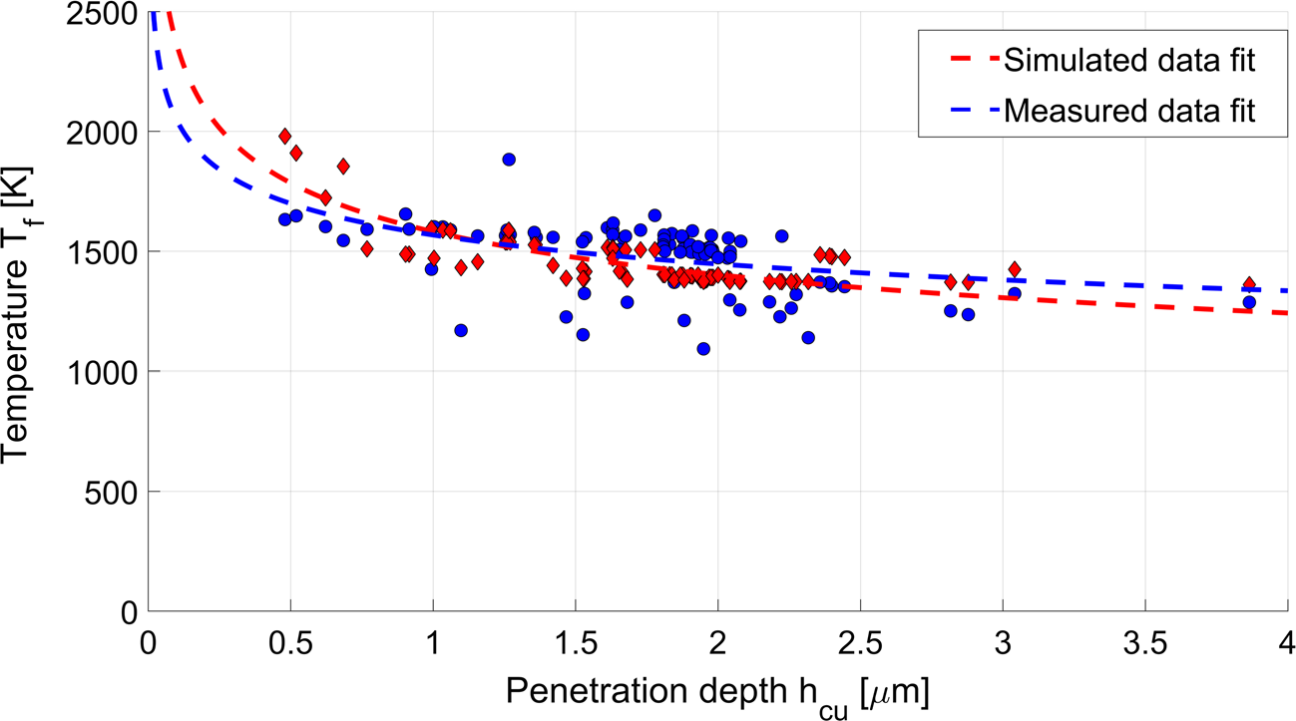
Comparison of the measured temperatures at single-grain scratch test and simulation results of the flash temperature model using Eq. 11

The data fit shown in Fig. 11 predicts very high temperatures exceeding 2000 K at very low penetration depths. The validity of the model and the fit for low penetration depths cannot be discussed with the experimental results since no observations for a penetrated depth lower than 500 μm are available. Since low penetration depths are accompanied by small contact areas; the diamond volume is relatively larger in comparison with the contact area and therefore has a relatively higher heat capacity. Heat generated in the sliding zone may therefore be distributed into the diamond more efficiently leading to overall lower flash temperatures than the model predicts. This may however be counteracted by the increasing heat generation due to plastic deformation associated with ductile removal at low cutting depth.

With increasing velocity, the Peclet number increases (see Eq. 3) and the workpiece surface temperature decreases in case of constant heat generation. Hence, at higher cutting speeds, where *Pe* ≤ 10, lower flash temperatures may be observed.

Last, it should be noted that consecutive scratches with the same grain lead to geometrical deviations due to wear as described in Section 2. The deviations are not considered in the calculation of the simulated data points. Wear may affect the width of the grain at a given penetration depth, which leads to an underestimation of the grain width as grains are typically tapered. Limitations of the experimental rig, where individual grains have to be isolated manually at the top of the pin, lead to a selection of large and blocky grains in the experiments. Analysis of the worn grains has shown that the ratio of the width to penetrated depth remains more or less constant.

Many recent approaches to modelling the workpiece surface quality in diamond wire sawing and grinding processes in general are based on the interaction between the workpiece and individual grains [34–36]. With improved methods and increasing computation power, larger surfaces can be simulated and improvement of the prediction requires the consideration of wear. The incorporation of a temperature model, which is able to predict temperature based on the contact geometry and the cutting velocity, is necessary to accurately model wear. Such a holistic approach has recently been presented [24].

## Conclusion

Experiments with solar-grade monocrystalline Si and diamonds electroplated onto commercial diamond wire as used in industrial diamond wire sawing applications have been conducted to study the flash temperature. For this purpose, a new test setup is introduced. In dry cutting conditions and for a contact length of 40 mm and cutting speed of *v*_*c*_ = 10 m/s, flash temperatures exceeding 1500 K have been observed.

A model for the calculation of flash temperatures when cutting Si with diamond has been derived and validated with experiments. The model considers heat flux in the diamond grain and binder, a heat source due to a sliding contact described by the tangential cutting force, and the sliding velocity through the Peclet number. Including a force model allows for the determination of flash temperature based on the cutting speed, the grain geometry, and the penetrated depth. This relationship may be used for kinematic process models for the diamond wire sawing process.

The flash temperature model and the experiments show that at lower depths of cut, with the presence of ductile material removal mode, high temperature is present at the grain tip. Moreover, as long as the ductile material removal is present, an increase in the depth of cut results in higher temperatures. As the depth of cut increases, material is removed in a more brittle regime, resulting in lower temperatures.

An empirically determined constant is necessary to compensate for deviations due to the simplifications adopted in the model development. For the assumptions employed, the constant has been determined as 4.5.

The model can be improved especially through the consideration of a more realistic contact area and modelling transient heat flow in the grain due to initial heating of the grain and with increasing binding temperature resulting from heat transfer to the wire. Further improvements may be achieved by taking heat generation through plastic deformation in the workpiece material into account.

## Data Availability

The data is being used for ongoing research and cannot be disclosed at the time.
